# Elimination of linezolid in patients undergoing low-flow continuous venovenous haemodiafiltration

**DOI:** 10.1186/cc10675

**Published:** 2012-03-20

**Authors:** T Ide, N Hori, Y Ikeda, K Takeda, S Nishi

**Affiliations:** 1 Hyogo College of Medicine, Nishinomiya City, Japan

## Introduction

It has been reported that linezolid (LZD) is highly removed in patients undergoing high-flow continuous venovenous haemofiltration (CVVH: blood flow and filtration rates were 186 ± 15 and 40 ± 8 ml/minute) compared with patients with normal renal function (NRF). It is generally considered that no adjustment of LZD dosage is needed in subjects undergoing CVVH. In Japan, continuous venovenous haemodiafiltration (CVVHDF) has preferentially been administered under low flow rate. Investigating the effects of flow rate on LZD removal during continuous renal replacement therapy is essential to regulate therapeutic dosages. We aimed to investigate the pharmacokinetics of LZD in CVVHDF patients in this setting.

## Methods

LZD (600 mg) was administered intravenously every 12 hours in ICU patients on CVVHDF and NRF patients (creatinine clearance 50 ml/minute). Blood and filtrate samples were collected at 0, 1, 1.5, 2, 3 and 5 hours after infusion from both groups. The elimination half-life (T_-1/2_), maximum concentration, concentration time curve (AUC), volume distribution (V_d_), clearance (CL) and sieving coefficient (Sc) were evaluated. Patient characteristics and CVVHDF parameters including the filter type, dialysate and filtration flow rates were recorded.

## Results

Fourteen CVVHDF patients and nine NRF patients were included into the study. CVVHDF was performed using polysulfone and triacetate membranes. Mean blood, dialysate and filtration flow rates were 79.3 ± 2.7 ml/minute, 8.7 ± 5.1 ml/minute and 5.5 ± 2.5 ml/minute, respectively. Sc was 0.86 ± 0.03. T_-1/2 _data (8.78 ± 3.74 vs. 5.54 ± 3.27 hours, *P *= 0.05) were significantly longer in the CVVHDF compared with the NRF group, AUC data (247.9 ± 107.8 vs. 136.0 ± 84.9 g hour/ml, *P *= 0.02) were significantly higher and CL (2.94 ± 1.38 vs. 5.92 ± 2.97 l/hour, *P *= 0.004) and V_d _(31.0 ± 3.8 vs. 35.8 ± 3.3 l, *P *= 0.01) data were significantly lower. LZD clearance was not correlated with the type of membrane used (polysulfone vs. triacetate: 2.8 ± 1.5 vs. 3.6 ± 1.2 l/hour, *P *= 0.39).

## Conclusion

Clearance of LZD in patients undergoing CVVHDF was significantly lower than in patients with normal renal function. Pharmacokinetic data from CVVHDF patients demonstrated that flow rates significantly influenced the efficiency of LZD removal. The maintenance dose of LZD may need to be reduced in patients undergoing CVVHDF under reduced flow conditions.
